# Nanoparticles for live cell microscopy: A surface-enhanced Raman scattering perspective

**DOI:** 10.1038/s41598-017-04066-0

**Published:** 2017-06-30

**Authors:** Maria Navas-Moreno, Majid Mehrpouyan, Tatyana Chernenko, Demet Candas, Ming Fan, Jian Jian Li, Ming Yan, James W. Chan

**Affiliations:** 1 University of California-Davis, Center for Biophotonics, Sacramento, 95817 USA; 2 BD Biosciences, San Jose, 95131 USA; 3 University of California-Davis, Dept. of Radiation Oncology, Sacramento, 95817 USA; 4 University of California-Davis, Dept. of Pathology and Laboratory Medicine, Sacramento, 95817 USA

## Abstract

Surface enhanced Raman scattering (SERS) nanoparticles are an attractive alternative to fluorescent probes for biological labeling because of their photostability and multiplexing capabilities. However, nanoparticle size, shape, and surface properties are known to affect nanoparticle-cell interactions. Other issues such as the formation of a protein corona and antibody multivalency interfere with the labeling properties of nanoparticle-antibody conjugates. Hence, it is important to consider these aspects in order to validate such conjugates for live cell imaging applications. Using SERS nanoparticles that target HER2 and CD44 in breast cancer cells, we demonstrate labeling of fixed cells with high specificity that correlates well with fluorescent labels. However, when labeling live cells to monitor surface biomarker expression and dynamics, the nanoparticles are rapidly uptaken by the cells and become compartmentalized into different cellular regions. This behavior is in stark contrast to that of fluorescent antibody conjugates. This study highlights the impact of nanoparticle internalization and trafficking on the ability to use SERS nanoparticle-antibody conjugates to monitor cell dynamics.

## Introduction

Therapy resistance as well as recurrence and metastasis are believed to be caused by a select group of cancer cells called cancer stem cells (CSCs) with significant ability to self-renew. In breast cancer, it has been shown that cells positive for CD44 and negative for CD24 are more aggressive, invasive, and tumorigenic^1^; and forced expression of HER2, in otherwise HER2-negative breast cancer cells, has been shown to enhance resistance to radiation therapy^2^. Based on this evidence, HER2^+^ , CD44^+^ and CD24^−/low^ has been proposed as a biomarker profile for the breast CSCs responsible for ionizing radiation (IR) therapy resistance^3^. However, the exact pathways enhancing cell survival, i.e., key factors controlling the overall pro-survival network after long-term irradiation, remain to be elucidated.

Studying the dynamics and evolution of biomarkers at the single cell level over multiple cell generations could greatly contribute to the understanding of breast CSC proliferation, thus helping identify the profile of the most aggressive CSCs. These studies could provide key information for the development of targeted therapy treatments to prevent and resensitize therapy resistant breast CSCs. Such studies would require antibody based labels with high multiplexing capabilities and photostability needed for long-term tracking of various antigen expression and fate over multiple cell divisions. However, conventional fluorescence based labels suffer from photobleaching and spectral crosstalk that limits multiplexing, making them unsuitable for such an application.

Nanoparticles (NPs) have very interesting properties that render them suitable for a broad range of applications, including cell imaging, *in-vivo* imaging, and sensing. In particular, surface enhanced Raman scattering (SERS) NPs have been extensively used to image fixed^4–9^, and live^10–16^ cells. From these studies, it is evident that SERS NPs provide clear advantages over fluorescence labeling in two important aspects: multiplexing and photostability^17^. Application of SERS NPs for dynamic live cell studies requires a careful consideration of the fundamental interactions between NPs and cells. Surprisingly, few studies using SERS NPs to image live cells consider the fact that living cells have a natural affinity for NPs, something that is well known in the fields of nanotoxicology and drug delivery. Cells are known to spontaneously uptake NPs depending on their shape and size^18–21^, and surface characteristics^22–24^. Moreover, targeted NPs, those that have been functionalized with monoclonal antibodies, have been shown to increase cellular uptake^25^ and multivalency, due to the presence of multiple antibodies on the surface of the nanoparticles, is known to affect nanoparticle-cell interactions^26^. For comprehensive reviews on the topic, see Yameen *et al*. and Kou *et al*.^27, 28^.

**Figure 1:**
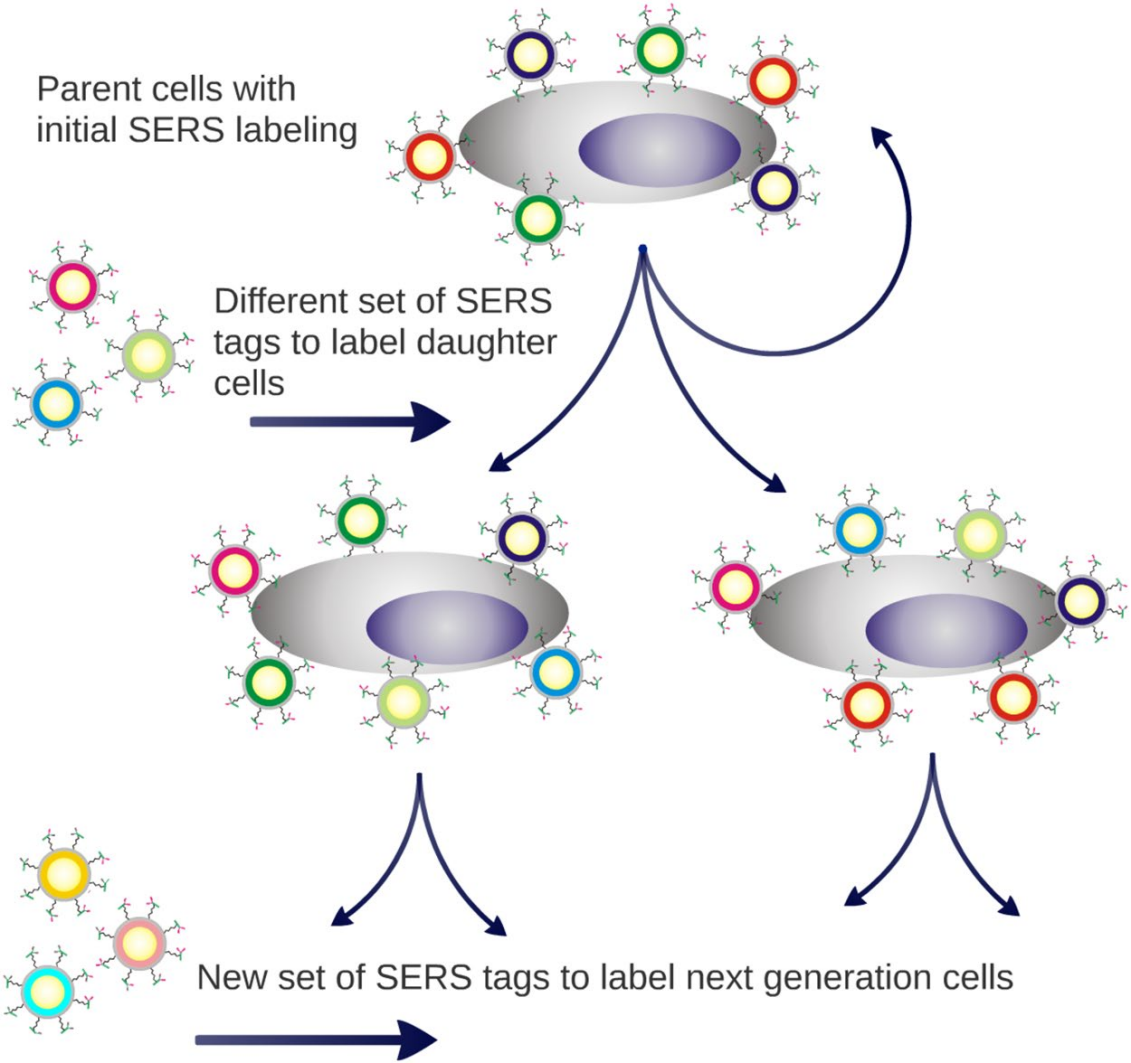
Schematic of the proposed approach for looking at the distribution of HER2 and CD44 inherited from parent to daughter cells, and also the newly expressed proteins. At each generation a different set of labels, represented by the different colors in the figure, would allow newly expressed and inherited proteins for each of the biomarkers of interest to be distinguished.

The original goal of this study was to take advantage of the non-photobleaching and multiplexing capabilities of SERS NPs to quantify the distribution of specific cell surface biomarkers (i.e., HER2 and CD44) and their dynamic distributions during cell cycle progression inherited from parent to daughter cells, as well as newly expressed proteins, to better characterize biomarker profiles during breast CSC proliferation (Fig. 1). The study presented here demonstrates that despite their known and appealing advantages, SERS NPs are not optimal for most live-cell imaging applications, such as monitoring dynamics of surface markers, due to NP internalization and trafficking by cells. Challenges and requirements for implementing SERS NPs in cell imaging and sensing applications are discussed.

## Results

### Cellular imaging with SERS nanoparticles

SERS NPs^29, 30^, were conjugated to HER2 and CD44 antibodies (Supplementary Fig. S1A) to investigate their labeling characteristics. Supplementary figures S1C and S1D show the Raman and absorption spectra of the NPs used in this study. Transmitted electron microscopy (TEM, Supplementary Fig. S1B) reveals that individual NPs may have more than one spherical gold nanoparticle encapsulated in the silica layer; thus, the NPs were differentially centrifuged to eliminate larger multi-core particles. The TEM results are confirmed with dynamic light scattering (DLS) measurements (Supplementary Fig. S1E) that indicate the NPs have a diameter distribution ranging from 100 to 400 nm. It is likely that larger diameters are due to NP aggregation rather than NPs having more than 3 gold cores.

To examine the labeling specificity of the SERS conjugates, we used darkfield microscopy to image various breast cancer cell lineages stained with HER2 or CD44 conjugated SERS NPs after fixation (Supplementary Fig. S2). The results in Fig. S2 correlate very well with the HER2 and CD44 expression levels as measured by western blot (Supplementary Fig. S3), indicating an excellent labeling specificity. Additionally, we performed dot blotting using a recombinant form of HER2 (rHER2) to further evaluate the specificity and sensitivity of the SERS conjugates. Supplementary Fig. S4 shows the results for the HER2 monoclonal antibody alone (Supplementary Fig. S4A) and for the SERS conjugate (Supplementary Fig. S4B) on mouse IgG (positive control), rabbit IgG (negative control) and rHER2. Dot blotting shows that the specificity of the antibody remains unaltered after conjugation to SERS NPs but the sensitivity to rHER2 showed a slight reduction demonstrated by the faint results obtained on dots other than the one with the maximum amount of rHER2 (50 ng).

**Figure 2:**
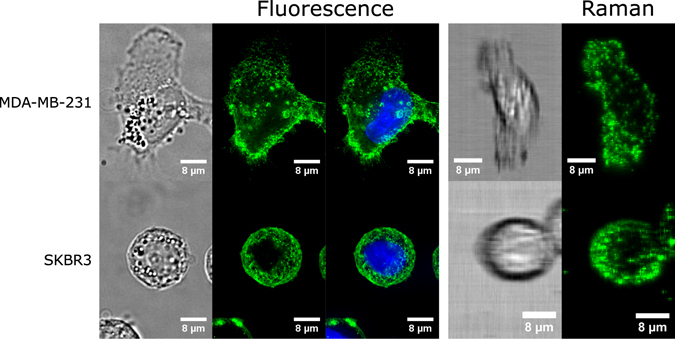
Side by side comparison of SERS and fluorescence images. Top: Fluorescence and Raman images of MDA-MB-231 cells stained with CD44 fluorescence and SERS conjugates, respectively. Bottom: Fluorescence and Raman images of SKBR3 cells stained with HER2 fluorescence and SERS conjugates, respectively. All images are accompanied by brightfield images for reference. Blue indicates nuclei of cells. Scale bars are 8 *μ*m.

The imaging properties of the SERS labels were evaluated using a custom built line-scan Raman microscope to acquire Raman images, which were then compared to their fluorescence counterparts. Figure 2 shows the maximum intensity projections of 3D Raman and fluorescence image stacks of SKBR3 and MDA-MB-231 cancer cells, which are HER2 positive and CD44 positive, respectively. Both SERS NPs and fluorophores were conjugated to the same clones of HER2 and CD44 antibodies, hence we expected very similar labeling characteristics. The images show that the SERS conjugates are able to label cells with the expected characteristics, and that the Raman images very much resemble their fluorescence counterparts, bearing in mind that the voxel size for the Raman images is 0.5 × 0.5 × 2 *μm* while the voxel size for the fluorescence images is 0.1 × 0.1 × 0.2 *μm*.

### Pulse-chase imaging and flow cytometry with SERS nanoparticles

**Figure 3:**
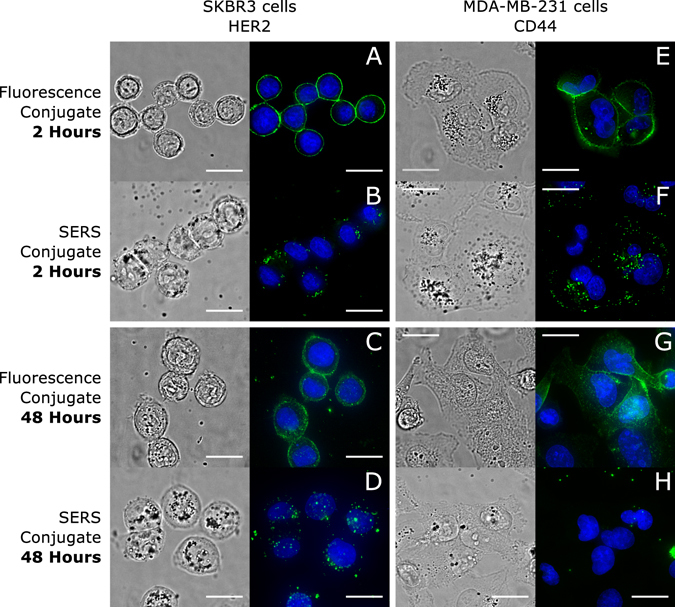
Nanoparticle labels are internalized and result in a different distribution than their fluorescent counterparts. (**A**) SKBR3 and (**E**) MDA-MB-231 cells incubated for 2 hours with HER2 and CD44 fluorescent conjugates, respectively. (**B**) SKBR3 and (**F**) MDA-MB-231 cells incubated for 2 hours with HER2 and CD44 SERS conjugates, respectively. (**C**) SKBR3 and (**G**) MDA-MB-231 cells incubated for 2 hours with HER2 and CD44 fluorescent conjugates, respectively, and allowed to incubate for additional 46 hours in fresh media. (**D**) SKBR3 and (**H**) MDA-MB-231 cells incubated for 2 hours with HER2 and CD44 SERS conjugates, respectively, and allowed to incubate for an additional 46 hours in fresh media. Blue indicates nuclei of cells. Scale bars are 20 *μ*m.

We performed initial pulse-chase experiments to characterize the behavior of the SERS conjugates and their interaction with cells. Figures 3A,B show fluorescence images (single optical plane) of SKBR3 cells incubated live for 2 hours with fluorescent and dual fluorescent-SERS HER2 conjugates, respectively (Full pulse-chase experiments images in Supplementary Figs. S5 and S6). The cells incubated with the fluorescent conjugate (Fig. 3A) show that, while some of the fluorophores show up intracellularly, most of the fluorescent signal remains on the cell membrane. Figure 3B, on the other hand, shows that the SERS conjugate is completely internalized and appears to be localized within cell compartments. Images for MDA-MB-231 cells incubated with CD44 conjugates, shown in Figs. 3E,F, exhibit similar behavior (Full pulse-chase experiment images in Supplementary Figs. S7 and S8).

SKBR3 and MDA-MB-231 cells incubated for 46 hrs with fresh media, after 2 hours incubation with HER2 and CD44 fluorescent conjugates, respectively, show images that resemble those obtained right after the initial incubation (Figs. 3C,G), thus implying that the fluorescent conjugates have very high affinity. More importantly, they are not being internalized. In contrast, images of the same SKBR3 cells incubated with HER2 SERS conjugates for 2 hours followed by incubation with fresh media for 46 hours show clusters of NPs that have been fully internalized (Fig. 3D). Images of MDA-MB-231 cells incubated under identical conditions with CD44 SERS conjugates (Fig. 3H) show very low numbers of NPs at the cells, thus demonstrating that the nanoparticle conjugates behave differently than their fluorescent counter parts. In fact, the results obtained with the HER2 fluorescent conjugate agree well with previous studies that show that HER2 is a receptor whose localization is restricted to the plasma membrane^31^. CD44 is also a receptor that localizes on the cell membrane, but unlike HER2, it is known to have a physiological response to certain antibodies^32^. Evaluation of the response of CD44 to the antibody clone is beyond the scope of this work, but it is important to consider the possibility of a downstream response of CD44 due to antibody binding.

**Figure 4:**
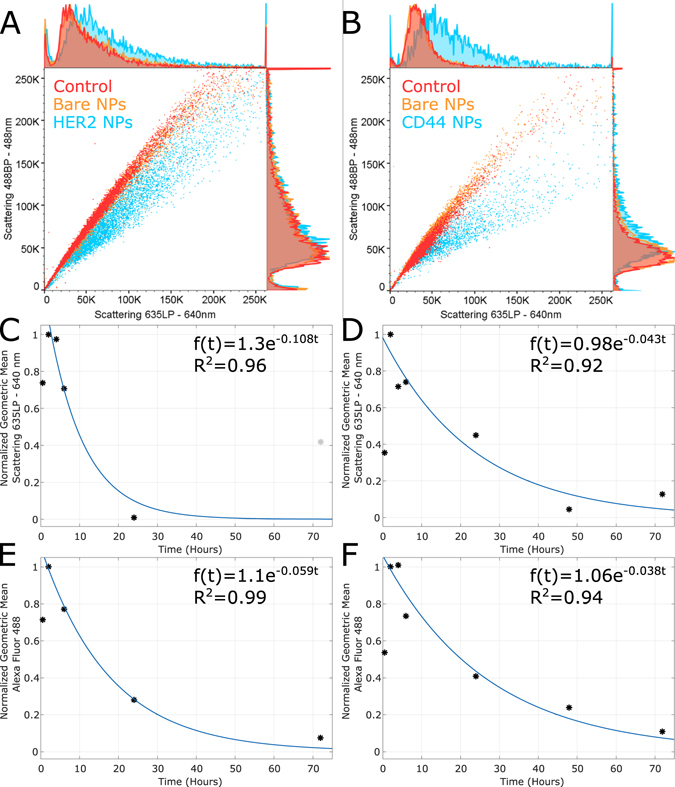
Nanoparticle cellular uptake and dynamics. Scatter plots of (**A**) SKBR3 and (**B**) MDA-MB-231 cells incubated with regular media (control), and media containing non-functionalized (bare) or functionalized nanoparticles. (**C**,**D**) The geometric means of the events distributions from the side scattering channel set by using a 623 nm filter and a 640 nm laser as a function of incubation time on cells incubated with HER2 and CD44 functionalized nanoparticles, respectively, and (**E**,**F**) from the Alexa Fluor 488 side scattering channel on cells incubated with HER2 and CD44 Alexa Fluor 488 conjugates, respectively. (**C**–**F**) Data has been normalized to t = 2 hours.

To further validate these observations, we carried out pulse-chase experiments where cells resuspended after fixation were analyzed using flow cytometry to detect scattering of 488 nm and 640 nm light, as well as the emission from Alexa Fluor 488. Figures 4A,B show the scatter plot of control cells, cells incubated for 6 hours with SERS conjugates (HER2 and CD44, respectively), and non-functionalized SERS NPs. The plots show that the cells incubated with non-functionalized NPs overlap with the control cells, indicating no uptake of these NPs. On the other hand, cells incubated with conjugated NPs show a significant shift relative to the control cells. Based on the absorption spectra of these NPs (Supplementary Fig. S1B), the change in the scatter plot is likely due to the slightly higher absorption in the green, which reduces the 488 nm scattering, but more importantly due to the increased light scattering in the red. This result confirms that NP uptake is specific to functionalized NPs.

Another set of pulse-chase experiments was carried out and either the scattering from the NPs or the emission from the Alexa Fluor 488 was quantified as a function of incubation time. Additionally, growth curves were generated (See methods). Cells were allowed to incubate for 2 hours with media that contained either the fluorescent or the SERS conjugate, at which point the cells were washed and allowed to incubate in fresh media for a total of 4, 6, 24 and 48 hours. Figures  4C,D show the geometric mean of the distribution obtained from the 640 nm side scattering channel as a function of incubation time for cells incubated with SERS conjugates, normalized to t = 2 hours. Figure 4E,F show the geometric mean of the distribution from the 488 nm channel (i.e., Alexa Fluor 488 emission) as a function of incubation time for cells incubated with fluorescent conjugates, also normalized to t = 2 hours. Here, it is important to note the rise in scattering or emission from 0.5 to 2 hours and the subsequent decay. For both types of measurements (i.e., scattering and fluorescence emission), the rise is due to the increase in binding of the conjugates. On the other hand, the decay has multiple contributions to it: 1) cell division, 2) unbinding of the conjugates given the finite affinity of the labels, 3) secretion after internalization, and in the case of scattering data, 4) intracellular redistribution of the nanoparticles, such as agglomeration inside organelles. While thorough understanding of the processes that led to the decay in the scattering data is important to better design nanoparticle-based imaging agents, we would like to draw attention to the differences in the decay rates of side scattering and fluorescence emission for cells incubated with nanoparticle and fluorescence conjugates, respectively. Defining *τ* as an e-fold time that accounts for both for all decay processes that are not cell division, the decay of emission of Alexa Fluor 488 or the scattering from the NPs can be expressed as *f*(*t*) = *f*_*o*_ *exp* − (1/*τ* + *ln*(2)/*t*_*cc*_)*t*. The doubling times *t*_*cc*_ for SKBR3 and MDA-MB-231 cells were estimated to be 41 and 26 hours, respectively (Supplementary Fig. S9 and methods). We find that *τ* in the case of the SERS conjugates is 2.2 and 1.5 times higher in SKBR3 and MDA-MB-231, respectively, compared to their fluorescence counterparts. This result supports the observations made from the imaging data that show a mismatch of the labeling characteristics observed with fluorescent and SERS conjugates.

### Intracellular distribution of fluorescence and SERS conjugates

We have shown that the dynamics of SERS conjugates differ significantly from that of fluorescence conjugates and what is expected from the known characteristics of HER2 or CD44. We wanted to obtain additional insight into the NP internalization pathways and intracellular localization. For this purpose, we performed pulse-chase experiments as described before. After fixation, cells were stained with antibodies that recognize proteins in the early endosomes (EEA1), late endosomes (Rab7), lysosomes (LAMP1) and Golgi apparatus (GM130), the most likely organelles where uptaken NPs could end up^33^. In these experiments, we also considered the possibility that the fluorophore or the SERS nanoparticles can become dissociated from the monoclonal antibody upon internalization.

**Figure 5:**
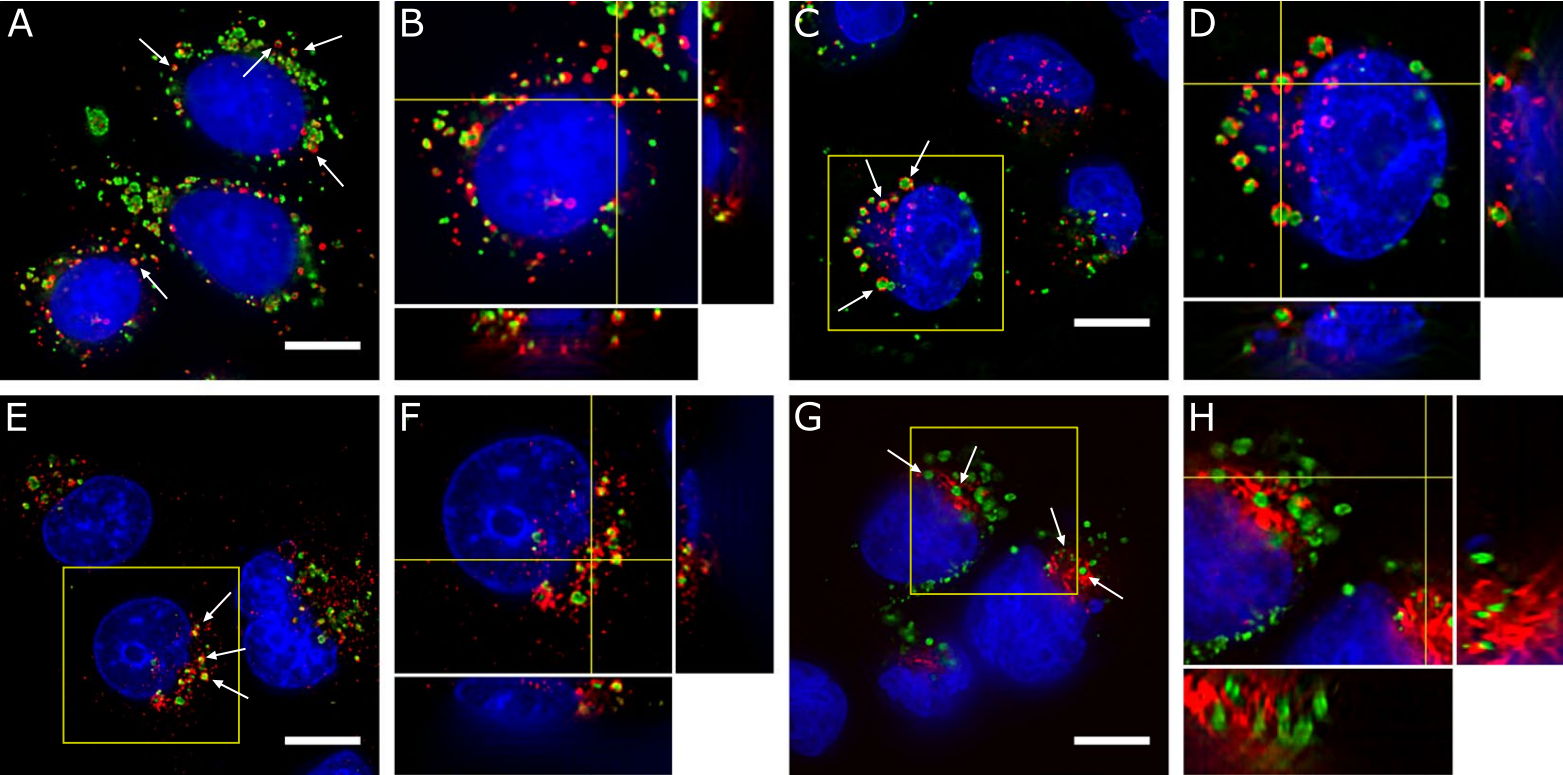
Intracellular distribution of nanoparticles. (**A**) SKBR3 cells incubated for 2 hour with HER2-nanoparticle conjugates (green) showing stained lysosomes (red). (**C**) MDA-MB-231 cells incubated with CD44-nanoparticle conjugates (green) for 2 hours and showing stained early endosomes (red). (**E**,**G**) MDA-MB-231 cells incubated for additional 2 hours in regular media and showing in red stained late endosomes and Golgi apparatus, respectively. (**B**,**D**,**F**,**H**) Orthogonal views of region of interest in shown in (**A**,**C**,**E**,**G**). Arrows and crosshairs point to examples of nanoparticles inside the various organelles. Nuclei shown in blue. Scale bars are 10 *μ*m.

In the case of SKBR3 cells exposed to HER2-SERS, only a few nanoparticles are localized to the early endosomes or the Golgi apparatus. By hour 4, some of the nanoparticles can already be seen inside lysosomes (Figs. 5A,B). See Supplementary Fig. S5 for the complete sets of images from cells incubated with HER2-SERS conjugates. As a control, organelles of cells incubated with Alexa Fluor 488-HER2 were stained (Supplementary Fig. S6). SKBR3 cells incubated with the HER2 fluorescence conjugate show little or no conjugate inside the cells, which once more demonstrates the differences in the imaging characteristics observed with SERS and fluorescence conjugates.

In MDA-MB-231 cells, CD44-SERS conjugates are clearly seen inside early endosomes during early time points (Figs. 5C,D), while also observed inside late endosomes and Golgi complex after 4 hours of incubation (Figs. 5E,F). After 6 hours, the CD44-SERS conjugates can also be seen inside lysosomes. See Supplementary Fig. S7 for the complete sets of images from cells incubated with CD44-SERS conjugates. As a control, organelles of cells incubated with Alexa Fluor 488-HER2 and -CD44 conjugates were stained (Supplementary Fig. S8). MDA-MB-231 cells show very low amounts of fluorescent CD44 conjugate inside the cells. The CD44-Alexa Fluor 488 that is internalized is found in endosomes, both early and late, as well as lysosomes. Albeit some internalization of the fluorescent conjugate occurs, live cell incubation of cells with SERS-CD44 and Alexa Fluor 488-CD44 conjugates still results in a remarkably different label distribution. The differences observed in both SKBR3 and MDA-MB-231 cells with the 2 types of conjugates can be attributed to different responses of either cell type to the NPs and/or different internalization pathways that are antibody dependent. A full understanding of the internalization pathways of these NPs conjugates warrants further investigation.

**Figure 6:**
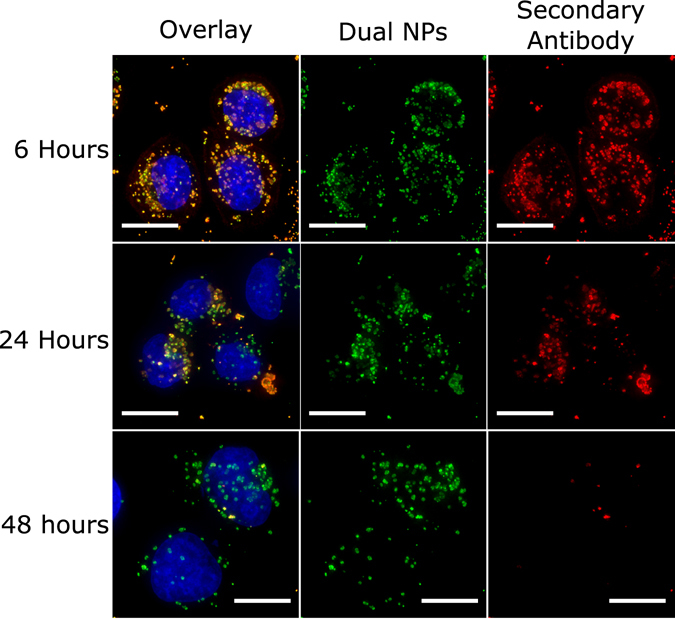
Conjugate degradation. Maximum intensity projections of SKBR3 cells incubated with dual SERS-fluorescence nanoparticles functionalized with HER2 (green) for 2 hours and additional 4, 22 and 46 hours with fresh media, for a total of 6, 24 and 48 hours incubation times. After incubation, HER2 antibody was counterstained with a secondary antibody (red). Cell nuclei are shown in blue. Scale bars are 10 *μ*m.

To evaluate the possibility that the antibody-nanoparticle conjugate could be modified upon intake, we incubated SKBR3 cells with dual SERS-fluorescence nanoparticles functionalized with a mouse HER2 antibody. At each time point, the cells were fixed, permeabilized and counter stained with an anti-mouse secondary antibody. Figure 6 shows the results at 6, 24 and 48 hours. For early time points we observe a perfect colocalization of the nanoparticles and the secondary antibody, thus showing that the HER2 antibody and the nanoparticle remained conjugated. After 24 hours, some nanoparticles do not show colocalization with the secondary antibody, and by 48 hours the secondary staining is almost absent. These results demonstrate that the antibody-nanoparticle conjugate is being dissociated upon internalization. Thus, after some period of time, the nanoparticles are no longer tracking HER2 as intended. As a control, we also counter stained cells incubated with a mouse HER2-Alexa Fluor 488 conjugate, with a mouse secondary antibody (Supplementary Fig. S10). We observe a perfect colocalization of both antibodies, hence demonstrating that the conjugate dissociation is exclusive to the NP construct.

## Discussion

Nanoparticles are very promising agents for multiple applications. For microscopic imaging, SERS nanoparticles offer an alternative to fluorescence with significant advantages such as photostability and high multiplexing capabilities. We demonstrated that on fixed cells our SERS conjugates are able to generate Raman images with the same characteristics as their fluorescent counterparts, and with excellent specificity. We intended to use the SERS nanoparticles to develop a framework that would allow us to image and monitor breast cancer biomarkers on live cells over multiple cell cycles. Instead, the nanoparticles show very interesting phenomena when interacting with live cells, but remarkably different than those observed with fluorescent labels. Pulse chase experiments using imaging and flow cytometry to follow the nanoparticle dynamics revealed that the cells uptake functionalized nanoparticles very efficiently and at a very fast rate, resulting in a label distribution that does not resemble that obtained with the fluorescent conjugates. We also found that nanoparticles functionalized with HER2 and CD44 antibodies have a different intracellular fate, with HER2-NPs quickly localizing to the lysosomes, and the CD44-NPs being more ubiquitously observed in endosomes and later on, inside the Golgi complex. Additionally, dual fluorescence-SERS nanoparticles functionalized with HER2 monoclonal antibody were shown to lose the antibodies upon internalization. Altogether, our results show that nanoparticles and fluorophore labels result in very different observations, thus rendering SERS NPs not suitable for the long term, live-cell monitoring of biomarker dynamics.

Our goal with this work is to demonstrate that the design of labels for live cell microscopic imaging has to be carefully considered and validated, particularly in the case of nanoparticles. There are issues with surface properties, size and shape of nanoparticles that may cause inadvertent effects. In addition, there is also the effects caused by antibody multivalency or protein corona formation, that could result in enhanced uptake or poor antibody binding. That is not to say that SERS nanoparticles are useless contrast agents. In fact, we demonstrated that they performed very well on fixed cells, that combined with their known incredible multiplexing capabilities^30^, renders them as outstanding reporters for flow cytometry applications. On the other hand, the fact that they are so efficiently and specifically uptaken by cells makes them excellent candidates for *in vivo* imaging applications, such as it has been demonstrated by Zavaleta *et al*.^30^ and Wang *et al*.^34^. Finally, another interesting application that takes advantage of nanoparticles cellular uptake is their use as non-targeted endocytic tracers. In this case, the nanoparticles are used to enhance the Raman signal from their immediate surroundings, thus reporting on their intracellular location, such as demonstrated by Kneipp *et al*.^35^, and more recently by Bando *et al*.^36^. Nanoparticles offer incredible opportunities for imaging and sensing applications, but careful consideration of all the aspects that mediate the cell-nanoparticle interaction is needed in order to take full advantage of their optical properties.

## Methods

### Materials

McCoy’s 5 A and RPMI-1640 cell culture media were purchased from ATCC (Manassas, VA, USA), Fetal bovine serum (FBS), MEM medium and MEM non-essential aminoacids from Corning (Tewksbury, MA, USA), prophylactic antibiotics (MycoZap Plus-CL) from Lonza (Basel, Switzerland). Bovine serum albumin (BSA), mouse IgG, rabbit IgG and paraformaldehyde (PFA) were purchased from Sigma Aldrich (St Louis, MO, USA). Anti-mouse IgG conjugated to Alexa Fluor 488 and Alexa Fluor 647, and Hoechst 33342 were purchased from Life Technologies (Grand Island, NY, USA). Glass-bottom 35 mm petri dishes were purchased from MatTek Corp. (Ashland, MA, USA). Rabbit antibodies that recognize HER2 (29D8), CD44(8E2), LAMP1 (D2D11), EEA1 (C45B10), Rab7 (D95F2) and GM130 (D6B1) were purchased from Cell Signaling (Danvers, MA, USA). Anti-Rabbit IgG conjugated to Rhodamine Red was purchased from Jackson ImmunoResearch Labs (West Grove, PA, USA). HER2 and CD44 recombinant proteins were purchased from Sino-Biological Inc. (Beijing, China).

### Nanoparticle and Fluorescence Antibody Conjugates

Nanoparticles consist of a 50 nm gold core encapsulated by a silica shell^29, 30^. Mouse anti-human HER2 (Neu 24.7) and CD44 (G44-26) monoclonal antibodies were conjugated to the nanoparticles using a PEG linker of 6 repeating units (Supplementary Fig. S1A). Monoclonal antibodies were also conjugated to Alexa Fluor 488, and Alexa Fluor 647. SERS and fluorescence conjugates were provided by BD Biosciences (San Jose, CA, USA).

### Cell Culture

MDA-MB-231 and BT-549 breast cancer cell lines were a gift from Dr. Jian Jian Li’s lab. SKBR3 breast cancer cells were purchased from ATCC (Manassas, VA, USA). MDA-MB-231 were grown in MEM medium supplemented with 10% FBS, 1% non-essential aminoacids and prophylactic antibiotics. BT-549 and SKBR3 cells were respectively grown in RPMI-1640 and McCoy’s 5 A media supplemented with 10% FBS and prophylactic antibiotics. Cells were incubated in a humidified atmosphere with 5% CO_2_ at 37 °C. Trypsin was used for cell passaging. Prior to staining and imaging, cells were seeded at a concentration of 2.5e4 cells/ml on glass-bottom 35 mm petri dishes and allowed to attach overnight.

### Cell Proliferation Test

Doubling rates were estimated from curves obtained by imaging glass bottom petri dishes at 0, 2, 4, 22, 46 and 70 hours seeded with the same number of cells (5*e*4 *cells*/*plate*). For each time point, 2 petri dishes were seeded. Cell nuclei were stained with Hoechst 33342, and each plate was imaged in 4 different regions. The images were processed according to the protocol presented by Busschots *et al*.^37^ and the number of nuclei were counted using ImageJ^38^. Reported values are the averages (± standard deviation) of the 8 images acquired for each timepoint and normalized to t0. The growth curves were modeled as *m*(*t*) = *A*_1_*Exp(−(1/*τ* − *ln*(2)/*t*_*cc*_)*t*, where *t*_*cc*_ is the doubling rate of the cells under normal conditions and *τ* is the time constant included to account for the effect of treatment. To deal with a linear case, we took the logarithm on both sides of the equation. To guarantee stationary condition^39^ we took the difference between the treated and the control series, and we tested the hypothesis of whether that difference is significantly different from zero using the method described by Santer *et al*.^40^. We set the significance threshold to *p* < 0.01.

### Immuno-staining on Fixed Cells

Cells were fixed in 4% PFA in 1× PBS for 10 min. Blocking was done in 5% BSA for 1 hour. Blocking solution was removed and monoclonal antibodies fluorescence (0.2 μg/ml) or SERS nanoparticles conjugates (10^10^ *nanoparticles*/*ml*) in 1% BSA were added and incubated at room temperature for 2 hours. When needed, samples were counter-stained with anti-mouse Alexa 488 (1:1000) or Alexa 647 (1:500) in 1% BSA for 30 min. Cells were stored in PBS at 4 °C until imaging. When permeabilization of membranes was necessary, it was done using 0.25% Triton X-100 (Sigma Aldrich. St Louis, MO, USA) for 5 minutes. In the case of organelle staining, samples were incubated with EEA1 (1:100), GM130 (1:1300), LAMP1 (1:200) and Rab7 (1:100) in 1% BSA for 2 hours and later counterstained with anti-rabbit Rhodamine Red (1:100) for 1 hour, both at room temperature. For antibodies against LAMP1 and Rab7 it was necessary to fix cells in 100% methanol at −20 °C for 15 minutes, the remaining of the protocol was the same as before.

### Immuno-Staining on Live Cells

No blocking step was taken. Cell growing media was removed and monoclonal antibodies fluorescence (0.2 μg/ml) or SERS nanoparticles conjugates (10^10^ *nanoparticles*/*ml*) in fresh growing media were added and incubated at 37 °C for 2 hours. Media was changed and cells were allowed to incubate for various time periods as specified in the main text. For imaging experiments, cells were grown on glass bottom petri dishes and fixed at each time point. For flow cytometry pulse-chase experiments, the cells were resuspended using Trypsin and then fixed in solution.

### Imaging

Fluorescence images were acquired using a Delta Vision deconvolution fluorescence microscope (Personal Deltavision, GE Life Sciences). Settings were adjusted so the entire dynamic range of the camera was utilized, and in the case of pulse-chase experiment were initially set for time point 0 and kept constant for the remaining time points.

Raman hyper-spectral images were acquired with a home-built line-scanning Raman microscope. The Raman microscope is equipped with a CW laser with a wavelength of 785 nm (Serval Plus, Sacher-Laser, Marburg, Germany) used as the excitation source. The laser beam is shaped into a line by means of a cylindrical lens (Thorlabs, Newton, NJ, USA) and confocality is ensured by a slit at the entrance of the spectrometer (Princeton Instruments, Trenton, NJ, USA). A 60×/1.2 NA oil immersion objective (Olympus) is used to focus the laser light onto the sample and to collect the Raman signal. A motorized stage (PRIOR Scientific, Rockland, MA, USA) is used for lateral scanning and a piezo objective scanner (PI, Karlsruhe, Germany) is used for Z positioning. Raman images are acquired with 0.54 *mW*/*μm* ^2^ power density at the sample, 0.8 sec. integration time per line and total scan time is 1–3 minutes per optical plane.

Darkfield (DF) images were acquired on a Leica DM-IRM using a 40×/0.55 NA objective with built-in DF illuminator and DF reflector and equipped with a CCD camera (CoolSnap EZ, Photometrics, AZ,USA)

### Flow Cytometry

Flow cytometry data were recorded using a BD LSRFortessa Cell Analyzer (BD Biosciences, San Jose, CA). The analyzer was set to acquire forward and side scattering off of the 488 nm laser using standard filters, and side scattering off of the 640 nm laser using a 632 nm long-pass filter. Data analysis was done using FlowJo software (FlowJo, LLC, USA)

### Dynamic Light Scattering and Absorption Spectra

The size and distribution of the nanoparticles were measured using dynamic light scattering (DLS) on a Zetatrac light scattering instrument (Microtrac, Inc., PA, USA). Data was analyzed using Microtrac Flex software (Microtrac, Inc., PA, USA). The mean sizes based on the area distribution (MA) were reported. Absorption spectra were recorded using a Varian Cary 50 Bio UV-VIS spectrometer (Agilent technologies, CA, USA). DLS and UV-VIS measurements were done at room temperature.

### Western Blotting

Immunoblotting was performed as described elsewhere^41^. Cellular extracts were loaded onto 5% and 10% SDS-polyacrylamide gels for HER2 and CD44 detection respectively. Protein was transferred to polyvinylidene difluoride membranes (Bio-Rad) using Bio-Rad semi-dry machine. Immunoblot analysis was visualized using the secondary antibody conjugated with horseradish peroxidase followed by the ECL Western blotting detection system (Amersham Biosciences, Little Chalfont, UK). Antibodies that recognize cytoplastic domain of HER2 (29D8) and CD44 (8E2) were purchased from Cell Signaling (Danvers, MA, USA)

### Dot Blotting

Solutions of HER2 recombinant protein, CD44 recombinant protein, mouse IgG and rabbit IgG at 3, 6, 12, 25 and 50 ng per microliter concentrations were dotted on nitrocellulose membranes (2 *μl* per dot). Membranes were allowed to dry for 5 min, blocked in 5% BSA for 1 hour, and incubated in primary antibodies or SERS-antibody conjugates for 2 hours at room temperature. Dot blot analysis was visualized using the secondary antibody conjugated with horseradish peroxidase followed by the ECL Western blotting detection system (Amersham Biosciences, Little Chalfont, UK).

## Electronic supplementary material

Supplementary Information
