# Supplementary material for: Nanoparticles for live cell microscopy: A surface-enhanced Raman scattering perspective
Source: Sci Rep. 2017 Jun 30;7:4471. doi: 10.1038/s41598-017-04066-0 (PMC5493633; doi:10.1038/s41598-017-04066-0)
Supplement: Supplementary file 1 — Supplementary Information [file 41598_2017_4066_MOESM1_ESM.pdf]

Supplementary Information
Nanoparticles for live cell microscopy: A surface-enhanced Raman scattering perspective

Maria Navas-Moreno[1], Majid Mehrpouyan[2], Tatyana Chernenko[2], Demet Candas[3], Ming Fan[3], Jian-Jian Li[3], Ming Yan[2], and James W. Chan[1,3,*]

[1]University of California-Davis, Center for Biophotonics, Sacramento, 95817, USA
[2]BD Biosciences, San Jose, 95131, USA
[3]University of California-Davis, Dept. of Radiation Oncology, Sacramento, 95817, USA
[4]University of California-Davis, Dept. of Pathology and Laboratory Medicine, Sacramento, 95817, USA
[*]jwjchan@ucdavis.edu

ABSTRACT

Surface enhanced Raman scattering (SERS) nanoparticles are an attractive alternative to fluorescent probes for biological labeling because of their photostability and multiplexing capabilities. However, nanoparticle size, shape, and surface properties are known to affect nanoparticle-cell interactions. Other issues such as the formation of a protein corona and antibody multivalency interfere with the labeling properties of nanoparticle-antibody conjugates. Hence, it is important to consider these aspects in order to validate such conjugates for live cell imaging applications. Using SERS nanoparticles that target HER2 and CD44 in breast cancer cells, we demonstrate labeling of fixed cells with high specificity that correlates well with fluorescent labels. However, when labeling live cells to monitor surface biomarker expression and dynamics, the nanoparticles are rapidly uptaken by the cells and become compartmentalized into different cellular regions. This behavior is in stark contrast to that of fluorescent antibody conjugates. This study highlights the impact of nanoparticle internalization and trafficking on the ability to use SERS nanoparticle-antibody conjugates to monitor cell dynamics.

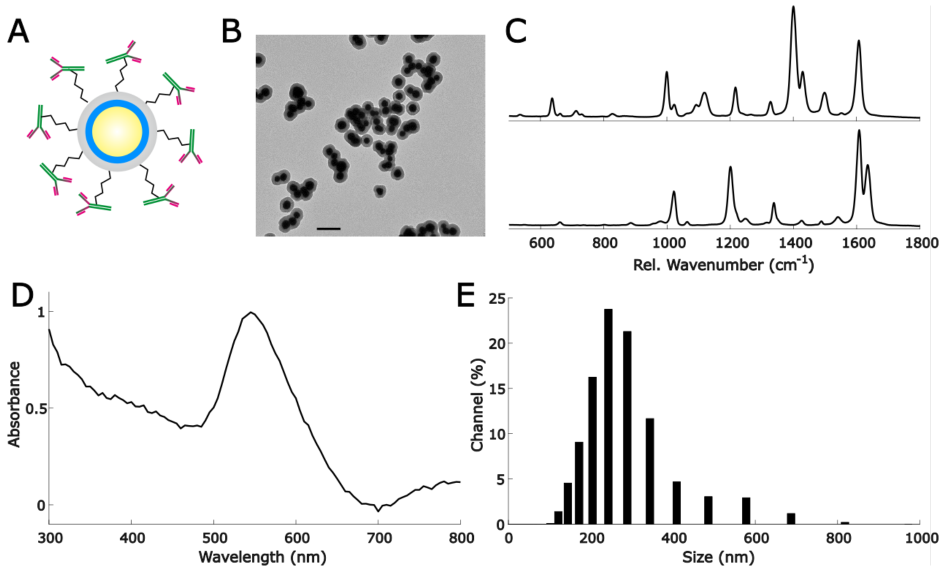

Figure S1. Properties of nanoparticles used in this study. (A) Schematic representation of the SERS NPs showing a gold core ($\tilde{6}0$ nm) coated with a layer of a Raman reporter (blue), and encapsulated in a silicon dioxide shell (gray). The antibodies (green) are attached to the nanoparticle via a PEG linker (black lines). (B) TEM (scale bar is 0.2 μm), (C) Raman spectra, (D) absorption spectrum, and (E) DLS size distribution.

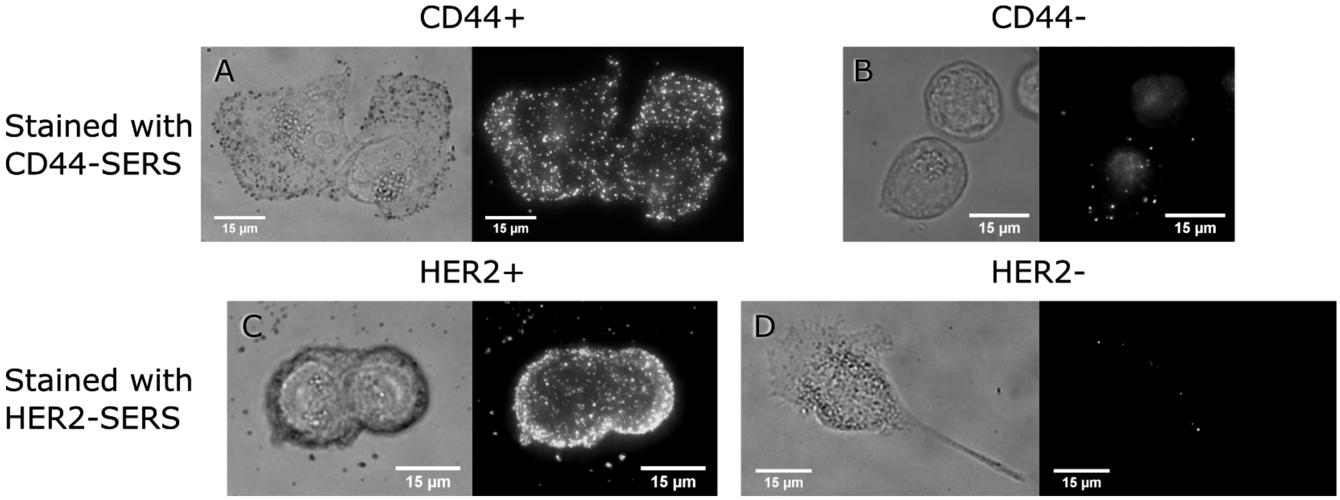

Figure S2. Darkfield microscopy on fixed cells, accompanied by brigthfield images, of cells stained with anti-CD44 SERS conjugates: (A) MDA-MB-231 (CD44+) and (B) SKBR3 (CD44-), and cells stained with anti-HER2 SERS conjugates: (C) SKBR3 (HER2+) and (D) BT-549 (HER2-).

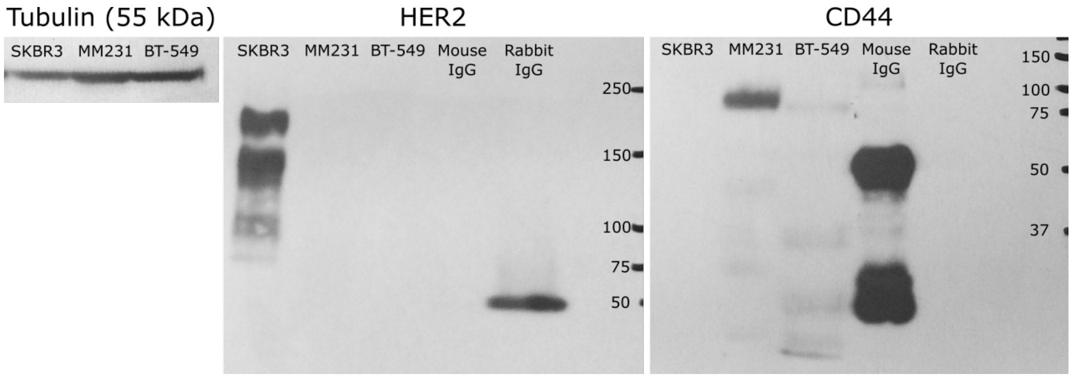

Figure S3. Western blot (WB) analysis on SKBR3, MDA-MB-231 (MM231) and BT-549 cell lineages of HER2 (using rabbit antibody) and CD44 (using mouse antibody).

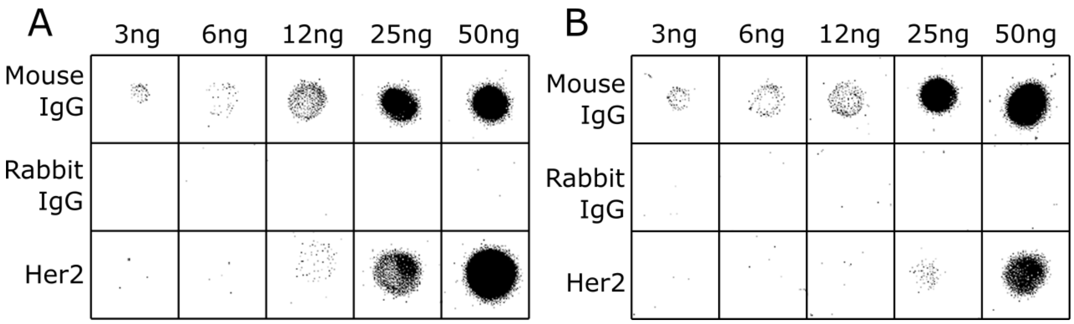

Figure S4. Dot blot analysis using recombinant HER2 protein for (A) anti-HER2 monoclonal antibody and (B) anti-HER2 SERS conjugate.

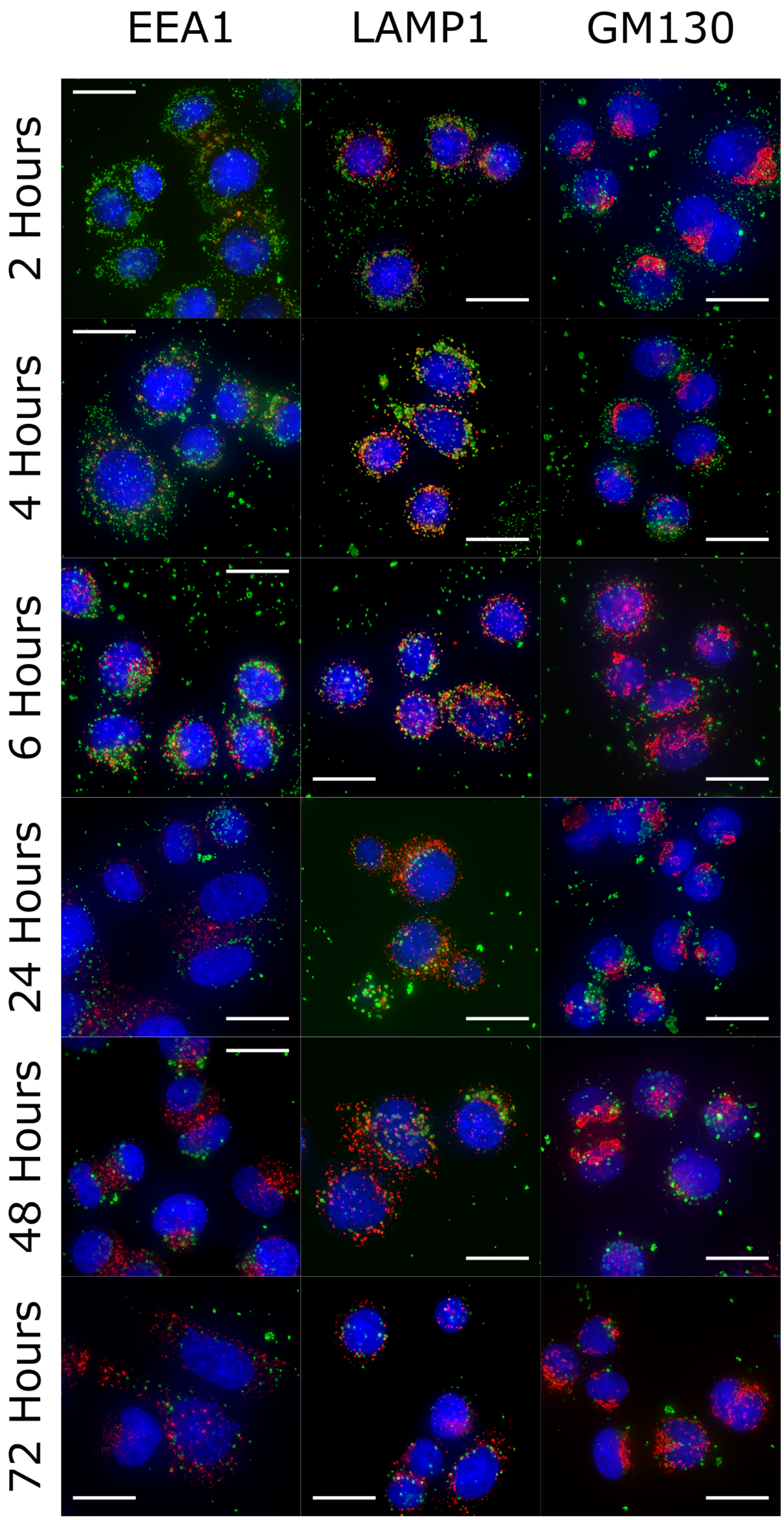

Figure S5. Representative fluorescence maximum intensity projections of SKBR3 cells incubated with HER2-SERS nanoparticles conjugate (shown in green) for 2 hours and then in fresh media for the remaining of the time indicated. Staining of early endosomes (EEA1), lyposomes (LAMP1) and the Golgi apparatus (GM130) are shown in red, and nuclei in blue. Images from 2 to 24 hours time points are single optical planes while those of 48 and 72 hours are maximum intensity projections.

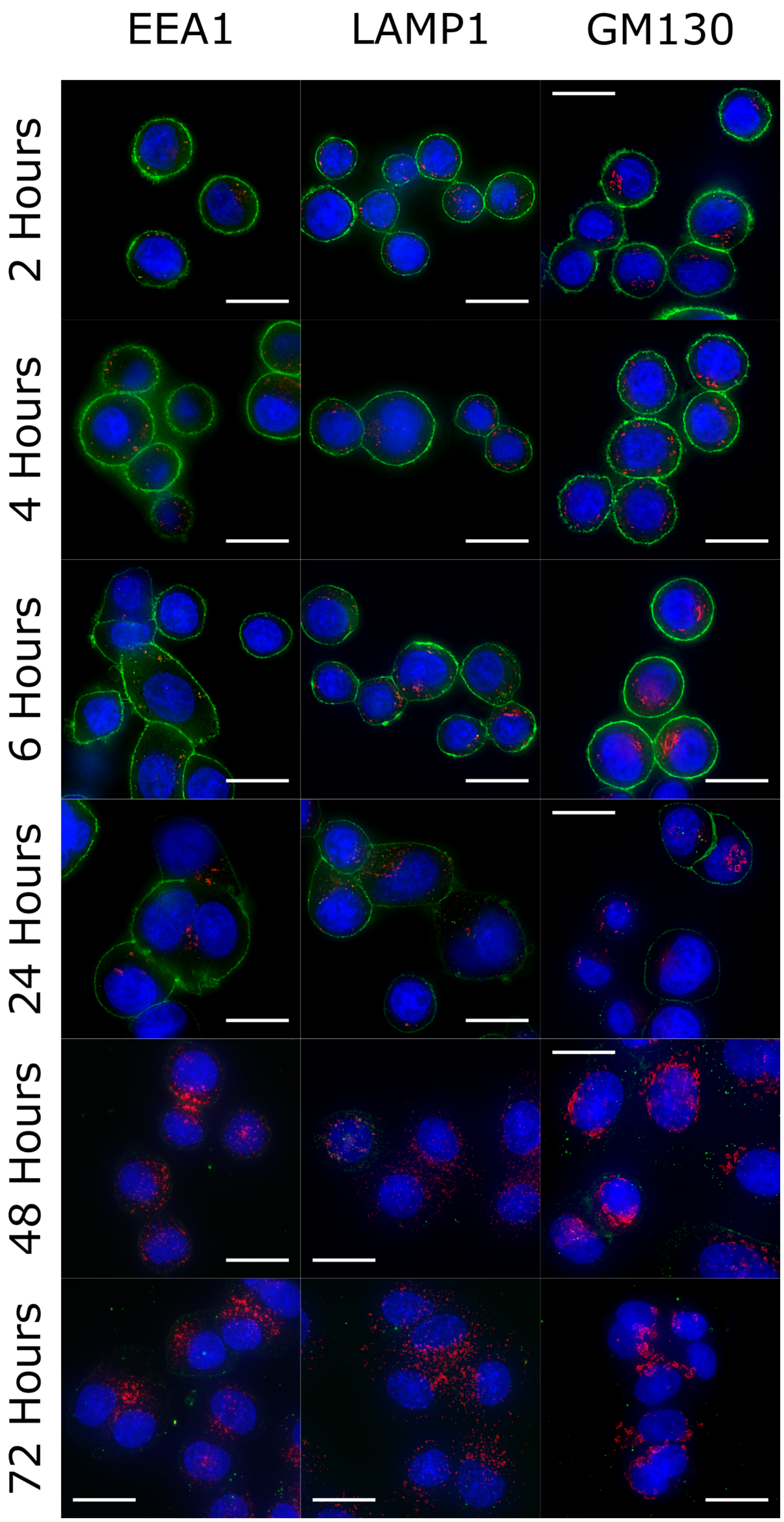

Figure S6. Representative fluorescence images of SKBR3 cells incubated with HER2-Alexa FLuor 488 conjugate (shown in green) for 2 hours and then in fresh media for the remaining of the time indicated. Staining of early endosomes (EEA1), lyposomes (LAMP1) and the Golgi apparatus (GM130) are shown in red, and nuclei in blue. Images from 2 to 24 hours time points are single optical planes while those of 48 and 72 hours are maximum intensity projections.

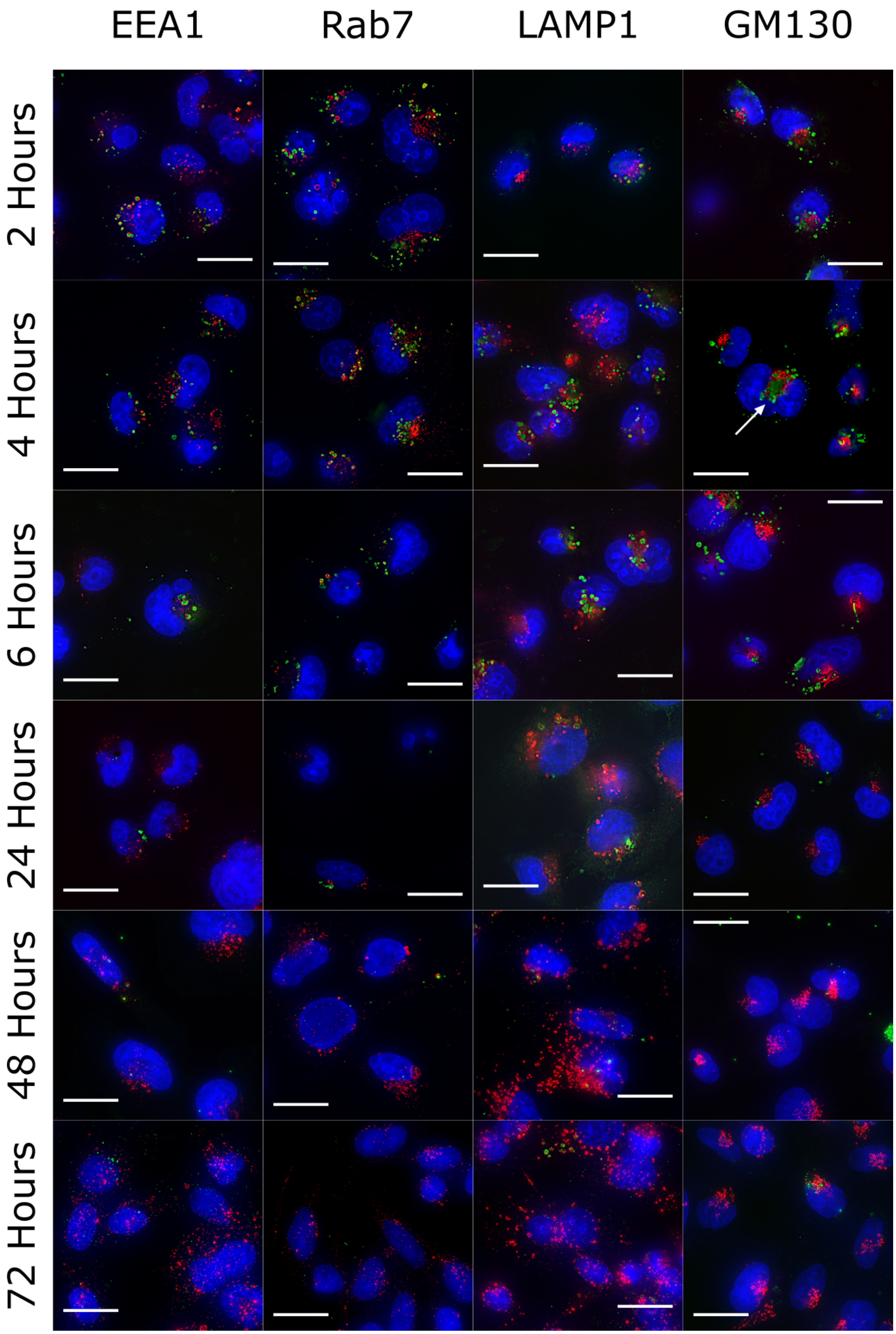

Figure S7. Representative fluorescence images (single optical plane) of MDA-MB-231 cells incubated with CD44-SERS conjugate (shown in green) for 2 hours and then in fresh media for the remaining of the time indicated. Staining of early endosomes (EEA1), late endosomes (RAb7), lyposomes (LAMP1) and the Golgi apparatus (GM130) are shown in red, and nuclei in blue.

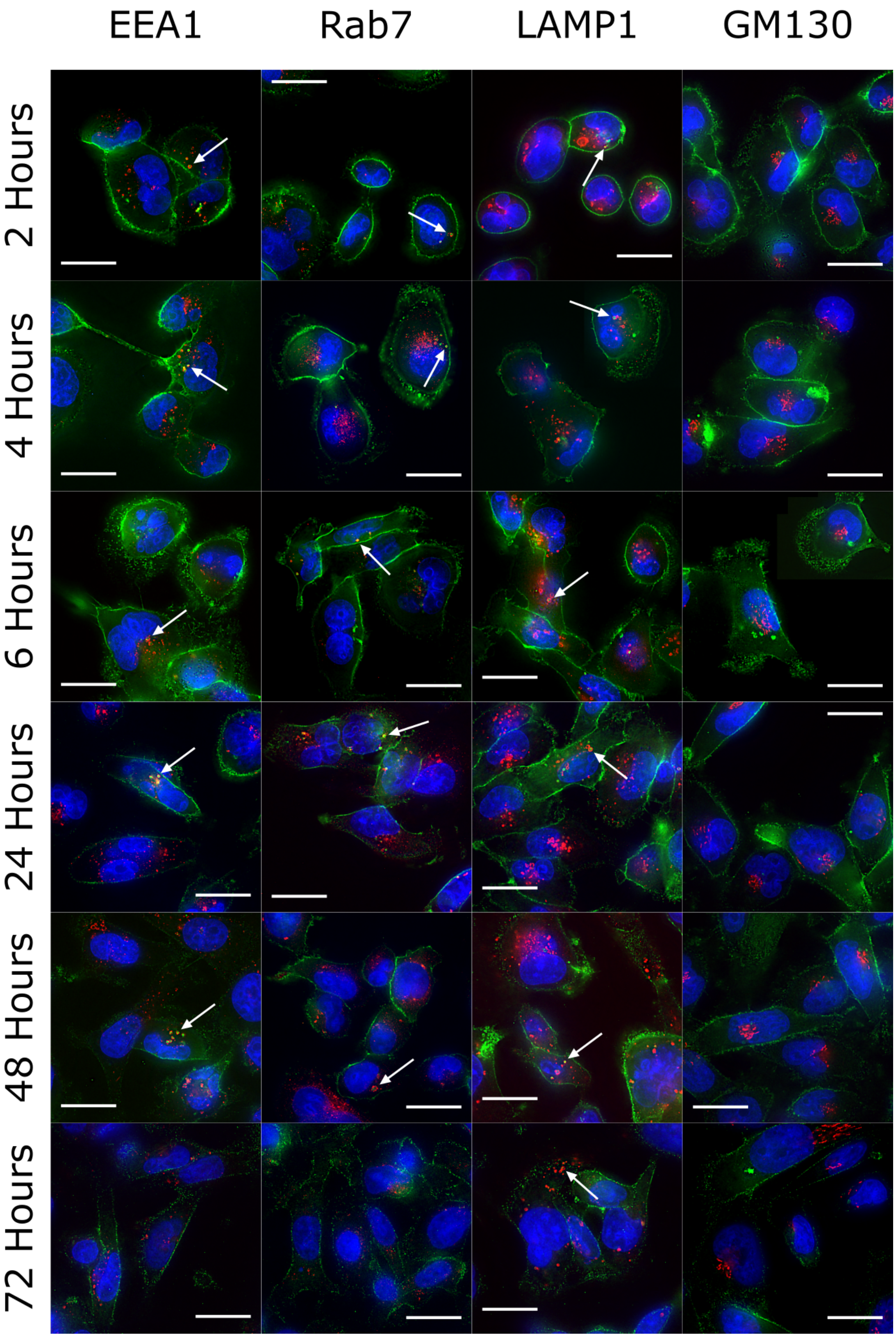

Figure S8. Representative fluorescence images (single optical plane) of MDA-MB-231 cells incubated with CD44-Alexa Fluor 488 conjugate (shown in green) for 2 hours and then in fresh media for the remaining of the time indicated. Staining of early endosomes (EEA1), late endosomes (RAb7), lyposomes (LAMP1) and the Golgi apparatus (GM130) are shown in red, and nuclei in blue.

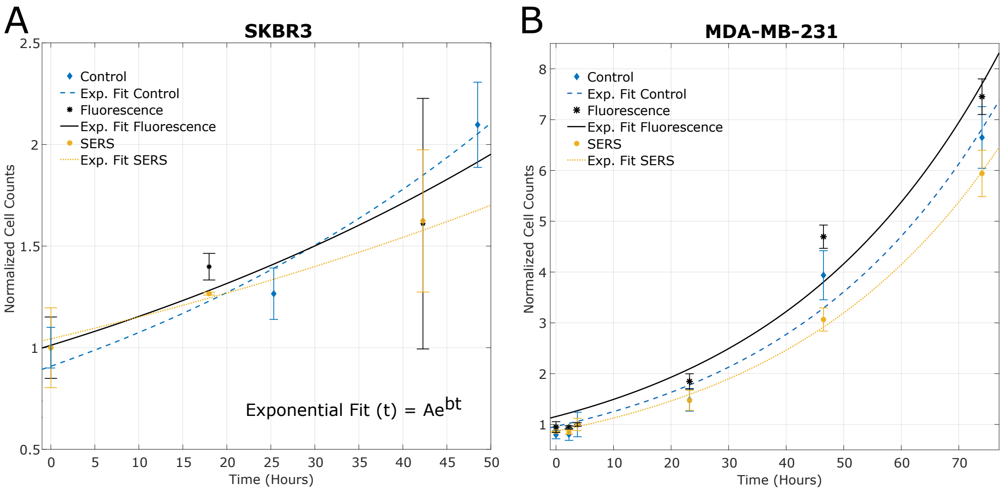

C

Cell Lineage	Control		Cells Incubated with SERS Conjugate		Cells Incubated with Fluorescence Conjugate	
	Exponential Parameter	Doubling Time (Hours)	Exponential Parameter	Doubling Time (Hours)	Exponential Parameter	Doubling Time (Hours)
SKBR3	0.0168	41.3	0.0097	71.5	0.01314	52.7
MDA-MB-231	0.0265	26.2	0.02604	27.1	0.02561	27.1

Figure S9. Growth curves for (A) SKBR3, and (B) MDA-MB-231, control cells (blue) and cells incubated with SERS (yellow) and fluorescence (black) conjugates. (C) Fitting parameter and doubling rates calculated for the curves presented in (A) and (B).

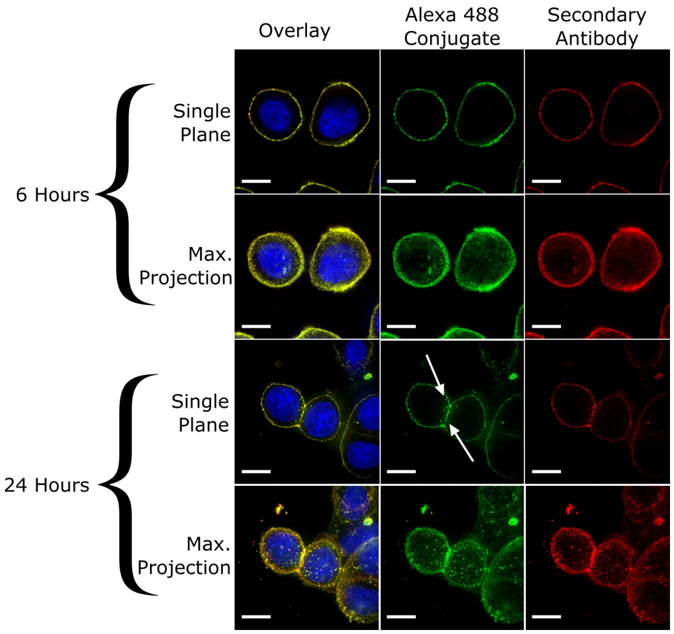

Figure S10. Single optical plane and maximum intensity projections of 3D fluorescence images stacks of SKBR3 Cells labeled with Alexa Fluor 488 conjugated to HER2 (green) for 2 hours and additional 4 and 22 with fresh media, for a total of 6 and 48 hours incubation times. After incubation HER2 antibody was counterstained with a secondary antibody (red). Cell nuclei are shown in blue. Scale bars are 10 μm.
